# *Ex Vivo* Perfusion-Simulation Measurements of Microbubbles as a Scattering Contrast Agent for Grating-Based X-Ray Dark-Field Imaging

**DOI:** 10.1371/journal.pone.0129512

**Published:** 2015-07-02

**Authors:** Astrid Velroyen, Martin Bech, Arne Tapfer, Andre Yaroshenko, Mark Müller, Philipp Paprottka, Michael Ingrisch, Clemens C. Cyran, Sigrid D. Auweter, Konstantin Nikolaou, Maximilian F. Reiser, Franz Pfeiffer

**Affiliations:** 1 Lehrstuhl für Biomedizinische Physik, Physik-Department & Institut für Medizintechnik, Technische Universität München, Garching, Germany; 2 Medical Radiation Physics, Lund University, Lund, Sweden; 3 Institute for Clinical Radiology, Ludwig-Maximilians-University Hospital Munich, Munich, Germany; Northwestern University Feinberg School of Medicine, UNITED STATES

## Abstract

The investigation of dedicated contrast agents for x-ray dark-field imaging, which exploits small-angle scattering at microstructures for contrast generation, is of strong interest in analogy to the common clinical use of high-atomic number contrast media in conventional attenuation-based imaging, since dark-field imaging has proven to provide complementary information. Therefore, agents consisting of gas bubbles, as used in ultrasound imaging for example, are of particular interest. In this work, we investigate an experimental contrast agent based on microbubbles consisting of a polyvinyl-alcohol shell with an iron oxide coating, which was originally developed for multimodal imaging and drug delivery. Its performance as a possible contrast medium for small-animal angiography was examined using a mouse carcass to realistically consider attenuating and scattering background signal. Subtraction images of dark field, phase contrast and attenuation were acquired for a concentration series of 100%, 10% and 1.3% to mimic different stages of dilution in the contrast agent in the blood vessel system. The images were compared to the gold-standard iodine-based contrast agent Solutrast, showing a good contrast improvement by microbubbles in dark-field imaging. This study proves the feasibility of microbubble-based dark-field contrast-enhancement in presence of scattering and attenuating mouse body structures like bone and fur. Therefore, it suggests a strong potential of the use of polymer-based microbubbles for small-animal dark-field angiography.

## Introduction

Complementary to conventional attenuation-based x-ray imaging, the generation of contrast from refraction and scattering that occur when x-rays pass through matter has gained strong interest in the last decades. As one of several possible approaches, grating-based interferometry has proven to be the most robust and practical in terms of translation from highly-brilliant x-ray synchrotron radiation sources to laboratory-based setups with conventional polychromatic x-ray sources, facilitating reasonable flux and relatively large fields of view [1, 2]. Therefore, grating-based interferometry shows great potential for possible implementation into a clinical environment, as supported by many *ex vivo* studies promoting improved soft-tissue contrast and complementary information obtained from grating-based phase contrast [3–7]. While the phase-contrast channel exploits refraction of x-rays in the sample in a range resolvable by the grating periods, Talbot-Lau interferometry also provides information about microstructures in the sample via an imaging channel commonly known as the dark field [2]. In contrast to feature depiction by attenuation or phase contrast, the dark-field signal even indicates structures below the resolution limit of the imaging system by measuring the strength of ultra-small-angle x-ray scattering in the sample [8–10]. Hence, grating-based interferometry yields three inherently registered contrast images, i.e. the attenuation-contrast image, the phase-contrast image and the dark-field image, each containing complementary information.

Recently, the technological developments in the area of x-ray phase-contrast and dark-field imaging (PCI, DFI) have advanced significantly such that *in vivo* projection imaging of mice has become possible [11]. Also, different issues standing in the way of translating PCI and DFI from bench to bedside, such as PCI at high photon energies [12], interferometry using a rotating gantry [13], or the usage of bent gratings for larger cone angles [14] have been tackled or—concerning grating manufacturing for larger fields of view—are under development. Even a first feasibility study for a clinically applicable PCI/DFI mammography setup has been conducted [15]. In the course of this general trend towards clinical applicability, naturally the question about dedicated contrast agents for this new technology arises. In conventional attenuation-based x-ray radiography and computed tomography (CT), the use of iodinated contrast agents represents the gold standard for many diagnostic questions, especially for vessel representation in projection angiography. As suggested by various applications of DFI, i.e. representation of changes of the alveolar structure in lungs [16, 17], microcalcifications in breast tissue [3, 18] and depiction of scattering at the trabecular structure of bones [19], an agent consisting of many interfaces between materials of strongly different indices of refraction is considered optimal for DFI. After the scattering properties of clinically used microbubble-based contrast agents widely applied in ultrasound imaging [20] were first investigated using diffraction enhanced imaging [21], we studied the potential for their use in Talbot-Lau interferometry in a preceding phantom study, confirming the ability of microbubbles to scatter x-rays at gas-to-shell interfaces [22]. Recently, Millard et al. [23] presented a Monte-Carlo simulation approach for a quantitative description of scattering due to bubbles. A comprehensive review about the wide variety in composition and properties of available microbubbles and their biomedical applications in imaging and drug delivery is given by Sirsi et al. [24].

In this study, we present an investigation of an experimental contrast agent based on microbubbles that are made up of a polyvinyl-alcohol (PVA) shell [25] with an iron-oxide coating [26]. The agent is under development for multiple purposes, such as magnetic resonance (MR), molecular imaging or ultrasound imaging (US) depending on the exact design parameters and functionalization of the surface (for example [27, 28]). Biocompatibility was shown [29] and is further supported by a recent study that successfully applies a subspecies of this new agent to *in vivo* rats for MR and Single Photon Emission CT (SPECT) [28]. To mimic conditions closer to the actual application to an animal, the PVA contrast agent was tested with the scattering background signal provided by a mouse carcass in DFI and compared to a clinically-used iodine-based contrast agent as a gold standard in attenuation-based imaging. Hereby, the potential for application of microbubbles in *in vivo* small-animal angiography was evaluated.

## Materials and Methods

### Contrast agents

Two different contrast agents were used in this study. The main focus lies on the magnetically coated microbubbles with the notation DMM150 by Surflay Nanotec GmbH, Berlin, Germany. The bubbles consist of a shell based on polyvinylalcohol (PVA), coated with four layers of super paramagnetic iron oxide (Fe_3_O_4_) nanoparticles (SPION). 100% PVA bubbles suspension corresponds to a concentration of 5 × 10^9^ bubbles/ml; in addition, the PVA bubbles were also measured diluted to 10% and 1.3% concentration. According to the manufacturer, the relative magnetization of this batch corresponds to 6.95 g iron per 10^9^ bubbles, resulting in an iron oxide amount of 9.6 × 10^−12^ g per bubble. The amount of PVA per bubble is reported by the manufacturer as 3.2 × 10^−12^ g. The thickness of the PVA shell lies between 400 and 600 nm. The SPION layers are coated onto the outside of the PVA shell by covalent coupling with alternating thin polyelectrolyte layers. The manufacturer determined the mean outer diameter of the bubbles by visual inspection via a microscope of a stained batch on 20 bubbles to be 5.5 ± 0.7 μm.

For gold standard comparison, the clinically used iodine-based x-ray contrast agent Solutrast^®^ 300 (Bracco Imaging Deutschland GmbH, Konstanz, Germany) was tested, pure and diluted to 10% volume concentration.

### Setup and image acquisition

The measurements were performed at a prototype preclinical dark-field and phase-contrast imaging scanner (developed in collaboration with *Bruker microCT*, Kontich, Belgium), described in detail by Tapfer et al. [13]. The setup in the scanner consists of a Talbot-Lau interferometer, an x-ray source and a detector installed on a gantry that can be rotated around the sample stage, facilitating *in vivo* imaging of small animals.

The interferometer comprises three gratings manufactured by *microworks*, Karlsruhe, Germany, optimized for a design energy of 23 keV. The experimental setup was described in detail in a previous publication by Velroyen et al. [22], and was used with an improved analyzer grating with deeper line structures (45 μm). The detector was operated in a 2×2 binning mode, resulting in an effective pixel size of 58 μm. The x-ray source has a tungsten target and was operated at maximum power (current of 525 μA) at the peak voltage of 35 kVp. Besides the source and phase grating, no additonal spectral filtering was applied.

To be able to extract absorption, phase-contrast and dark-field images, phase stepping was used [2, 30]. For a preliminary contrast comparison, a reference sample of pure water and the pure PVA bubble suspension were measured in identical plastic vials of 4.2 mm inner diameter (in the cylindrical part).

Each scan had 5 phase steps with 3.33 s exposure, resulting in a total exposure time of 16.65 s for one set of images of all three modalities.

For the main experiment, a mouse carcass was used to gain a proper representation of background signal in terms of bone structure and fur for contrast-agent testing. (According to the German Animal Welfare Act, TSchG §7 subsection 2(3), sacrificing an animal for the use of its organs or tissue for scientific purposes does not fall under the definition of an animal experiment. Therefore such an *ex vivo* experiment as presented here does not require a dedicated ethics approval.) The mouse was obtained through standard breeding and housing at Helmholtz-Zentrum Munich. It was sacrificed by an authorized expert via CO_2_ overdose. To be able to reliably locate the contrast-medium position, a plastic tube with an inner diameter of 1.6 mm was surgically implanted underneath the peritoneum entering at the chest and exiting in the lower abdominal area. The tube was fixated using thin sewing threads. The experimental protocol used with PVA bubbles (100%, 10%, 1.3%) and Solutrast (100%, 10%) started with the injection of 0.3 to 0.4 ml of contrast agent, so that the main part of the implanted tube was filled with contrast medium.

In each experiment the carcass was imaged twice: once with contrast agent in the tube, and once with water filling the tube. Both sets were taken with 10 steps and 5 s exposure time per step. The accumulated dose corresponds to approximately 5.7 mGy for both sets together. The dose had been determined via measurement of the dose rate at the according tube voltage using a patient skin dosimeter (Unfors PSD, Unfors Instruments AB, Billdal, Sweden) placed in the center of a polymer cylinder with a diameter of 3 cm as an appropriate mouse phantom.

To check that the bubbles from the measured batches are intact, a small amount was imaged using a Zeiss Axiovert Light Microscope with 20-fold magnification lens.

### Subtraction image processing

Prior to subtraction of the non-enhanced from the contrast-enhanced scan, the three complementary image signals transmission, dark-field and differential phase were extracted from the raw phase stepping sets.

The opening of the specimen chamber and the attachment of the syringe in between the two scans (with and without contrast agent) could introduce movement of carcass and tube. To reduce the resulting artifacts in the subtraction images, a simple rigid registration procedure was performed: The non-enhanced and the contrast-enhanced images were shifted relative to each other, and, if necessary, one was slightly magnified or demagnified, to find the maximum correlation between the two scans. After this improved registration, the non-enhanced image was subtracted from the contrast-enhanced image so that an image only containing residual noise and the signal created by the contrast agent was obtained.

Towards lower contrast-agent concentrations, the contrast agent signal started to enter the regime of statistical image noise, but was still visually distinguishable. For a clearer discrimination, in addition to the raw subtraction signal, a second representation of the subtraction image was created by further re-binning by a factor of two and additional means of noise reduction: First, to isolate the true contrast-agent induced signal, any signal, that would appear outside of the sample area (i.e. where the transmission is around unity) was masked out. Second, due to the deep grating structures, the cone-beam geometry of the setup causes shadowing in regions far from the optical axis [14] leading to fewer counts, increased noise and reduced visibility. Thus, the areas near the left and right margins of the field of view were masked out using a visibility map from a reference scan. Third, individual median-filtering was applied to reduce noise and enhance connected areas. Subsequently the masked and median-filtered signal was displayed by a hot color scheme superimposed on the original transmission image in the areas where a threshold was reached. The individual parameters for filter-kernel size and threshold were chosen based on best visual appearance and feature clarity and are as follows: PVA 100% 5 pixels squared, 0.05 threshold; PVA 10% 9 pixels squared, 0.025 threshold; PVA 1.3% 9 pixels squared, 0.015 threshold; Solutrast 100% 5 pixels squared, 0.05 threshold; Solutrast 10% 9 pixels squared, 0.015 threshold.

## Results

**Fig 1:**
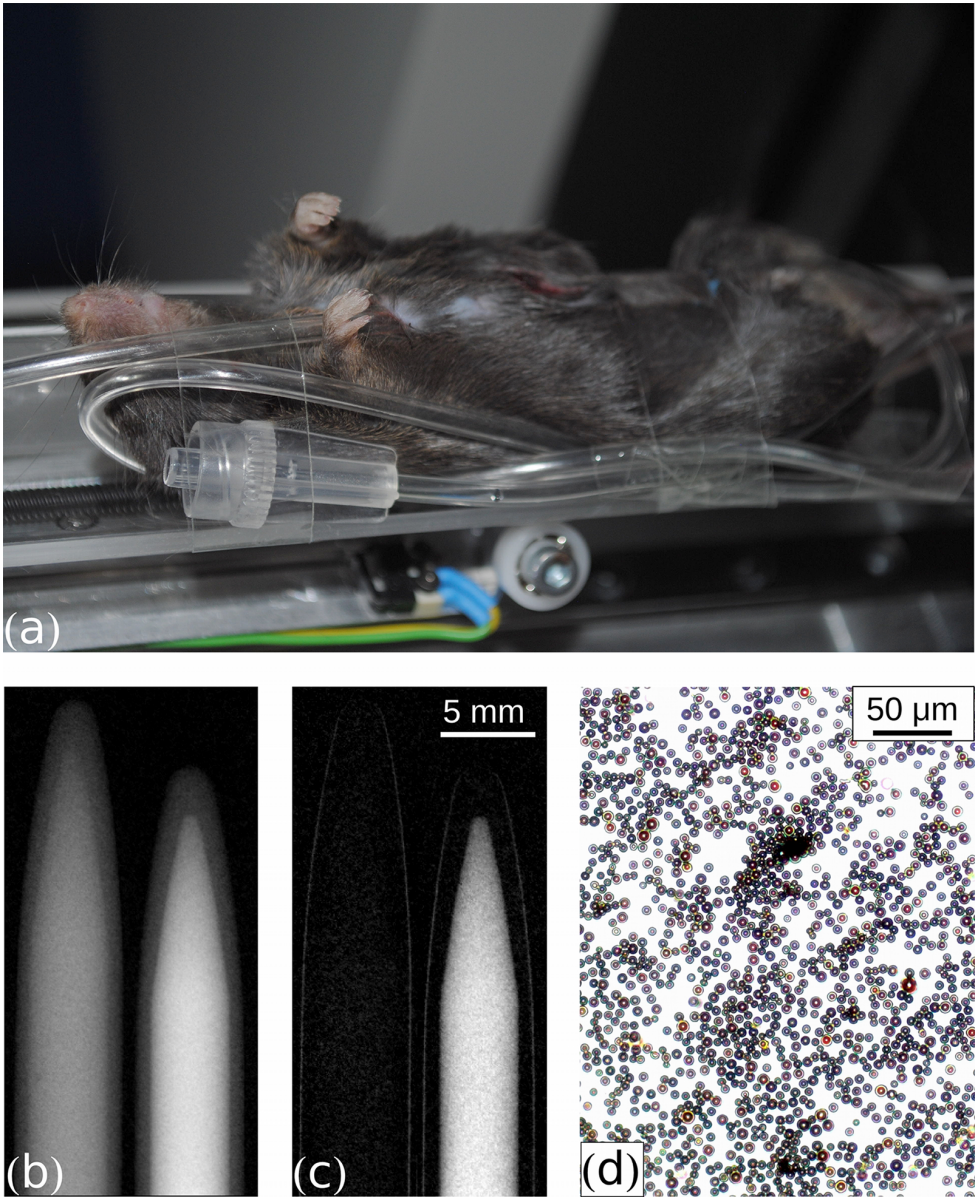
Sample positioning and preliminary imaging of PVA contrast agent. (a) Mouse carcass fixated on the animal bed of a preclinical phase-contrast and dark-field CT scanner. A plastic tube was surgically inserted underneath the peritoneum and some spare volume of the tube for flushing the contrast agent downwards was attached to the right side of the mouse. (b) Transmission image of plastic vials containing water (left) and PVA microbubbles (right). Grey values ranging from 0 to 0.35. (c) Dark-field image of plastic vials containing water (left) and PVA microbubbles (right). Grey values ranging from 0 to 0.75. (d) Visible-light brightfield microscopy image of PVA microbubbles.

*In vitro* images of the magnetically coated PVA bubbles and a water reference sample are shown in Fig 1(b) and 1(c). The PVA bubbles clearly show a stronger dark-field signal than water. Transmission is also reduced by the coated PVA bubbles, but this reduction is not as strong as in the dark-field signal. Note that, as common in radiology, in the presented transmission images black corresponds to high transmission and white to low transmission. In case of dark field, black codes low scattering, whereas white depicts strong scattering. Since the PVA bubbles had not been fixated, accumulation of the bubbles at the upper surface of the round vial (i.e. along the vertical axis of the containers) was not averted. Because of the resulting inhomogeneous bubble distribution a reliable quantitative analysis of the signal cannot be performed. However, these images serve to deliver a qualitative impression of the difference in x-ray scattering power between pure water and coated PVA bubbles. We refrained from a fixation or gelification in order not to alter the chemical and osmotic environment and risk bubble destruction. To check if intact bubbles are present in the sample, small amounts of the suspensions were extracted and inspected via visible-light microscopy: Fig 1(d) shows intact bubbles.

**Fig 2:**
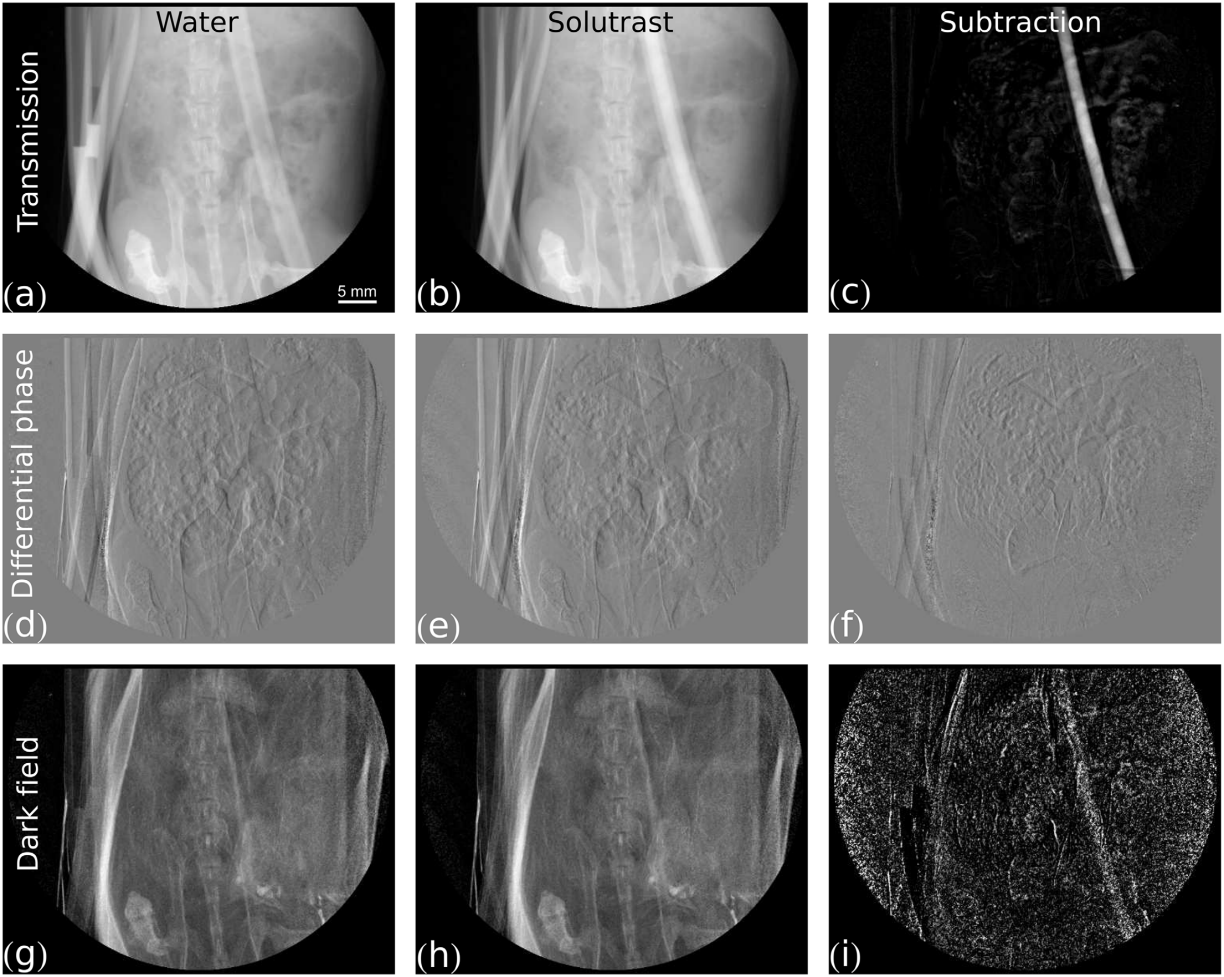
Measurement of pure Solutrast contrast agent. Grey value scaling is given in brackets. The first column shows the same image with the tube filled with water for three different contrast modalities: (a) transmission [0, 0.9], (d) differential phase [$-\frac{\pi }{2}$, $\frac{\pi }{2}$], (g) dark field [0, 1.0]. The second columns shows the same image with the tube filled with pure Solutrast in three different modalities: (b) transmission [0, 0.9], (e) differential phase [$-\frac{\pi }{2}$, $\frac{\pi }{2}$], (h) dark field [0, 1.0]. The third column shows the subtraction of the image with contrast agent (second column) from the image with water (first column) for three different contrast modalities: (c) transmission [0, 0.2], (f) differential phase [$-\frac{\pi }{2}$, $\frac{\pi }{2}$], (i) dark field [0, 0.13].

**Fig 3:**
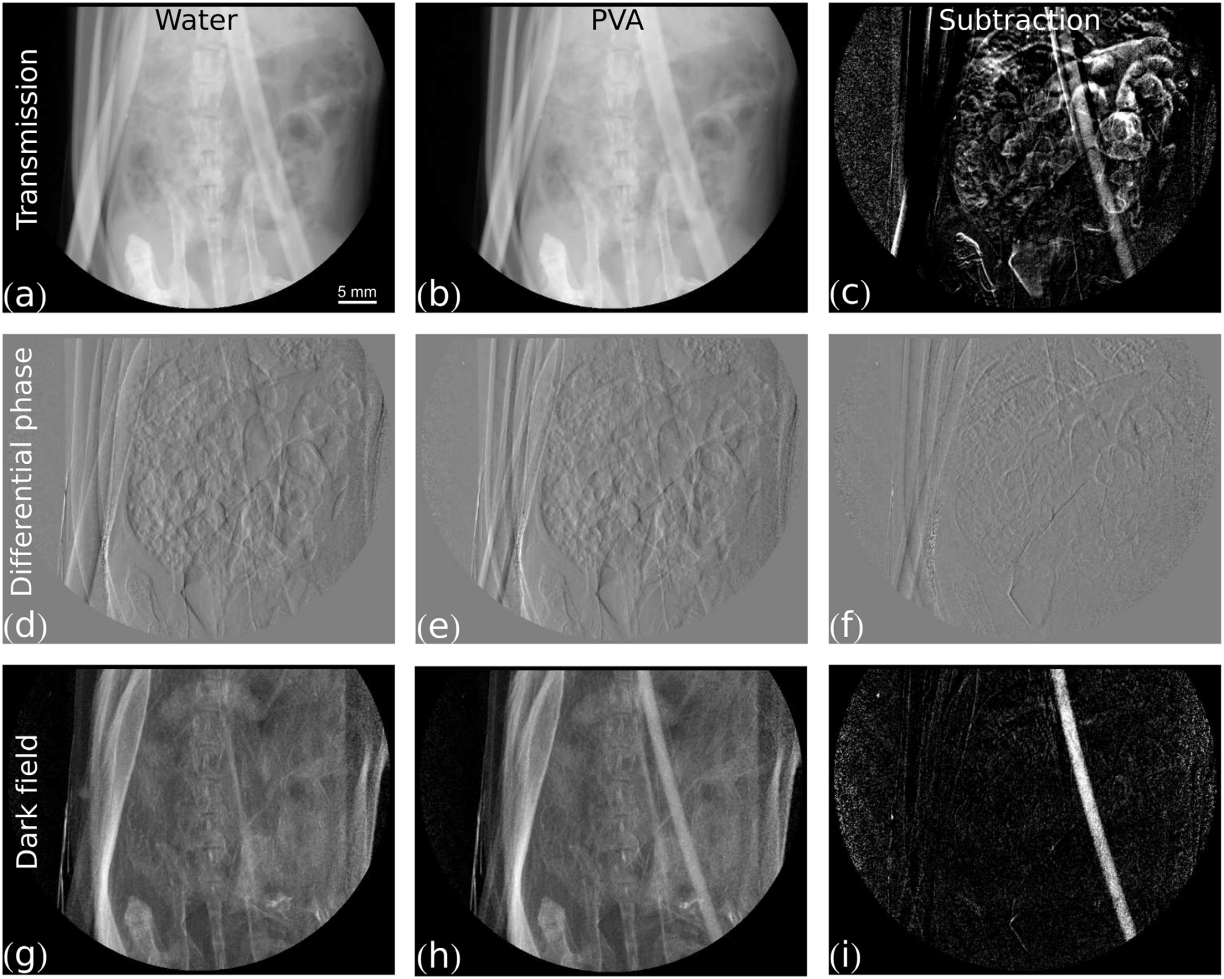
Measurement of pure PVA bubbles contrast agent. Grey value scaling is given in brackets. The first column shows the same image with the tube filled with water for three different contrast modalities: (a) transmission [0, 0.9], (d) differential phase [$-\frac{\pi }{2}$, $\frac{\pi }{2}$], (g) dark field [0, 1.0]. The second column shows the same image with the tube filled with pure PVA-microbubbles suspension in three different modalities: (b) transmission [0, 0.9], (e) differential phase [$-\frac{\pi }{2}$, $\frac{\pi }{2}$], (h) dark field [0, 1.0]. The third column shows the subtraction of the image with contrast agent (second column) from the image with water (first column) for three different contrast modalities: (c) transmission [0, 0.04], (f) differential phase [$-\frac{\pi }{2}$, $\frac{\pi }{2}$], (i) dark field [0, 0.3].

**Fig 4:**
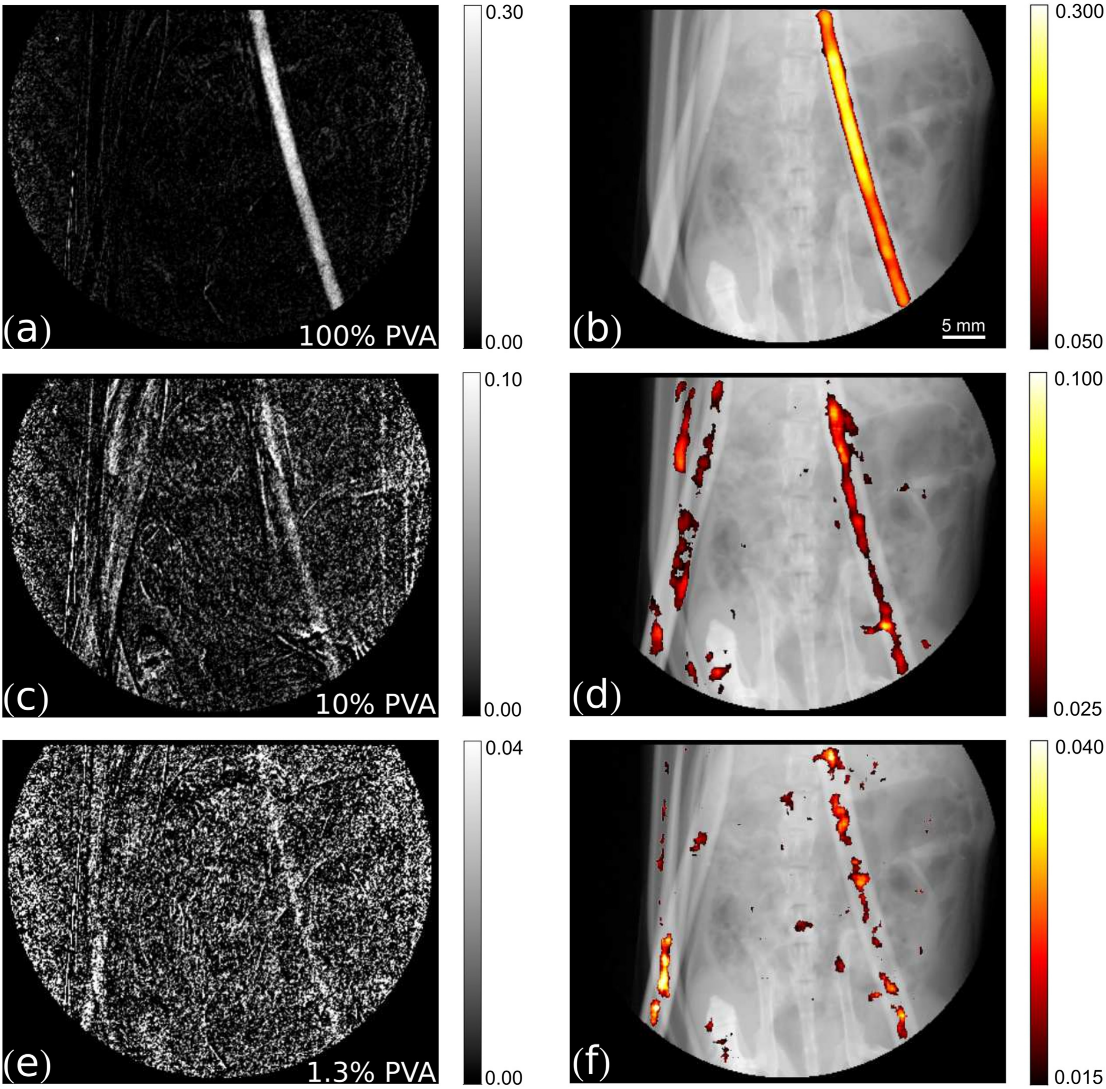
Dark-field signal subtraction and multimodal images. Left column: dark-field signal subtraction images. Right column: threshold-limited, filtered superimposition of the dark-field subtraction image (in color) on the respective original transmission image (grey values range: [0, 0.9]). (a)-(b) 100% PVA microbubbles suspension, (c)-(d) 10% PVA microbubbles suspension, (e)-(f) 1.3% PVA microbubbles suspension.

**Fig 5:**
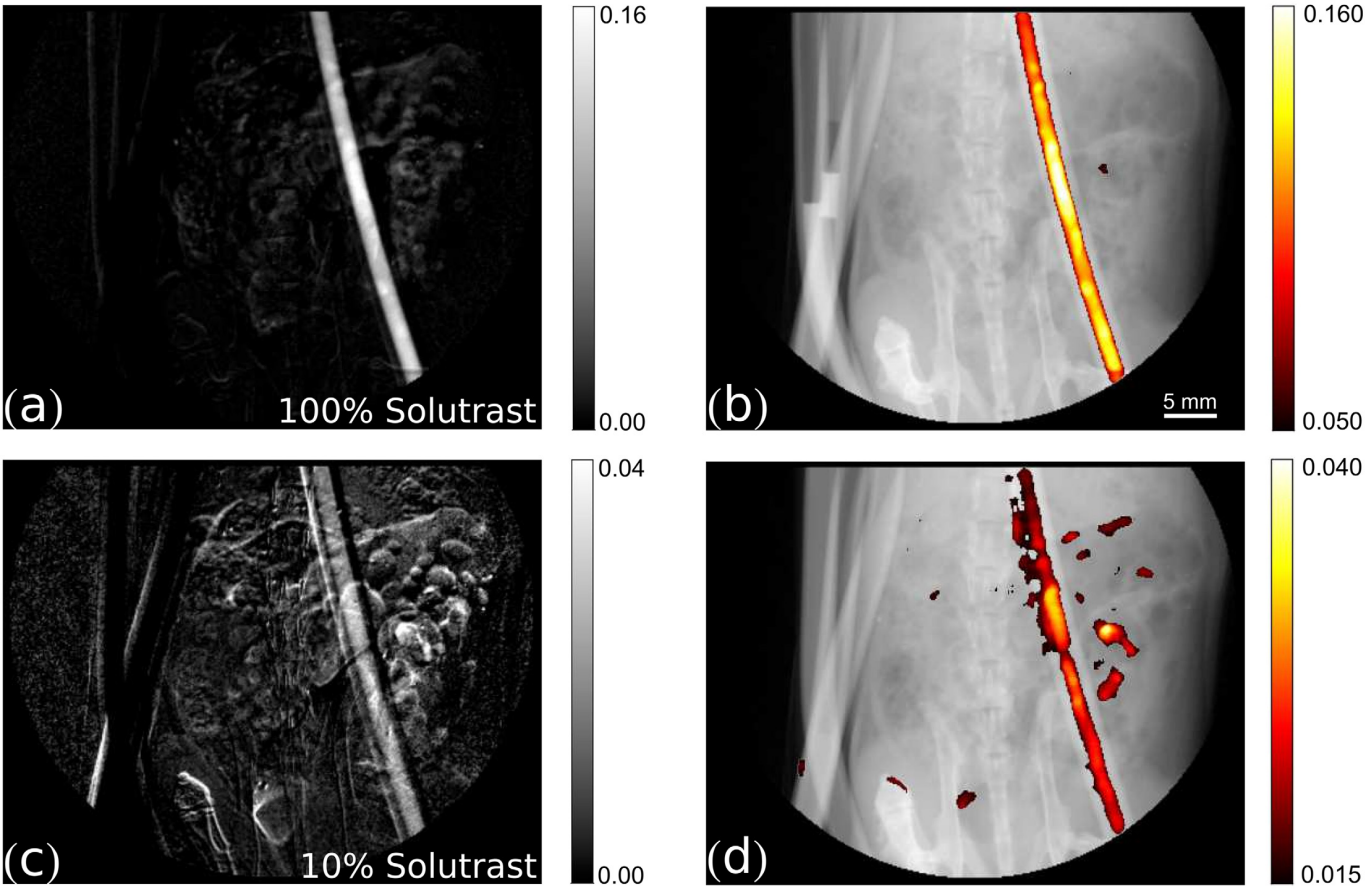
Transmission signal subtraction images. Left column: transmission signal subtraction images. Right column: threshold-limited, filtered superimposition of the transmission subtraction image (in color) on the respective original transmission image (grey values range: [0, 0.9]). (a)-(b) 100% Solutrast, (c)-(d) 10% Solutrast.

The results of the subtraction imaging series are shown in Figs 2–5. As expected, the pure, clinically available iodine-based Solutrast strongly absorbs x-rays, such that in the transmission image in Fig 2(b) the contrast-agent filled tube is clearly visible. The subtraction image in Fig 2c shows that the signal strength is well above the noise floor. In Fig 2(a) the tube inserted underneath the peritoneum is filled with water. Since this image was acquired after the one in Fig 2(b), the contrast agent that was pushed onward is visible in the spare tube part positioned next to the carcass on the left margin of the image. These left-over contrast agent portions from prior measurements are the reason why also in some of the following subtraction images signal enhancement can appear in this area. For the sake of completeness, Fig 2(d)–2(f) show the differential phase signal of the measurement with water and the one with Solutrast in the tube, as well as the subtraction image. They demonstrate that, due to the differential nature of the phase signal, the subtraction is dominated by movement of the spare tube and evolving gas bubbles in the intestines. Fig 2(g)–2(i) show the dark-field signal of the acquisition with water, with pure Solutrast and their subtraction. The contrast-agent containing tube hardly shows up in the direct dark-field image, but is visible in the subtraction image. However, the generated dark-field signal is weaker than the transmission signal, since Solutrast is a homogeneous fluid. In fact, the occurring reduction in visibility, which leads to the visible dark-field signal, can most likely be attributed to beam-hardening of the x-ray spectrum. Since the visibility of the interferometric image is strongly dependent on the energy of the x-rays, it can decrease if the more optimal lower energies are filtered out by the sample [31], i.e. the contrast agent, resulting in an enhanced dark-field signal.

Fig 3 shows the corresponding non-enhanced, contrast-enhanced, and subtraction images for all three modalities for the pure, SPION-coated PVA bubbles. Contrary to the Solutrast measurement, in the case of PVA bubbles, the dark-field signal generated by the bubbles in the tube is sufficiently strong to clearly stand out in the contrast-enhanced dark-field image (Fig 3(h)). The subtraction image in Fig 3(i) shows a signal strength well above the noise floor. On the other hand, the absorption in the coated shell generates a visible signal in transmission (Fig 3(b) and 3(c)), which, however, is weaker than the dark-field signal. In fact the transmission subtraction image is dominated by gassing in the intestines. As in Fig 2, the differential phase signal does not show a relevant change by the contrast agent (Fig 3(d)–3(f)).

Though the pure PVA bubble contrast agent used in Fig 3(i) is not compatible with *in vivo* in mice, other animals (such as e.g. rats) are tolerant to this high dose of magnetite-coated PVA microbubbles, and images could be obtained under the assumption that the imaging system would be fast enough to capture the first pass of a bolus injection into a large vessel. Therefore, contrast-enhanced subtraction imaging with lower PVA bubble concentrations was performed. Fig 4 shows the dark-field subtraction images of 100%, 10% and 1.3% PVA bubbles concentration in comparison, as well as the respective filtered subtraction signal superimposed on the original transmission image for an improved anatomical orientation. Although the signal strength of the contrast-agent filled tube reaches the noise level when the bubble concentration is reduced, the human eye can still discern the tube-shaped true signal from the noise floor even for the lowest measured concentration. The overlaid and filtered signals in hot color scheme in Fig 4(d) and Fig 4(f) show that, besides the true signal in the tube of interest, only the signal in the spare volume of the tube, fully explicable by residual contrast agent from the prior contrast-agent shot with higher concentration, and some smaller noise patches due to residual motion artifacts light up in the mouse anatomy. These results indicate the feasibility of contrast-enhanced perfusion dark-field imaging of the larger vessels in small animals.

For a complete comparison with the gold-standard iodine contrast agent, the corresponding transmission subtraction images and the filtered, superimposed signals of the 100% and 10% concentration of Solutrast are shown in Fig 5. Here, the main contribution to the residual signal in the filtered images, besides the true signal in the tube, is due to movement of the tube and evolving gas in the abdominal area of the mouse.

## Discussion

In a preceding *in vitro* study on clinically established ultrasound microbubbles [22], the albumin-based Optison [32] had been identified as a promising candidate for contrast-enhanced dark-field imaging. Unfortunately, a quantitatively reliable comparison between Optison and the PVA bubbles, which would be of strong interest for benchmarking, was not possible, because Optison has been taken off market and is not even available for research-only purposes. It is known that microbubbles based on lipid/albumin shells are known to be less stable than polymer-based bubbles [27], and that unfavorable osmotic conditions can effect bubble size or even destroy them [33]. The Optison we had on stock from our previous study turned out to have a reduced scattering power and consequently Optison is not included in this study.

Please note that the maximum concentration of PVA bubbles presented here is one order of magnitude larger than the concentration that Optison was available at. Concentration along with *in vivo* tolerance can be assumed to be of high importance in terms of contrast agent design for dark-field imaging.

**Fig 6:**
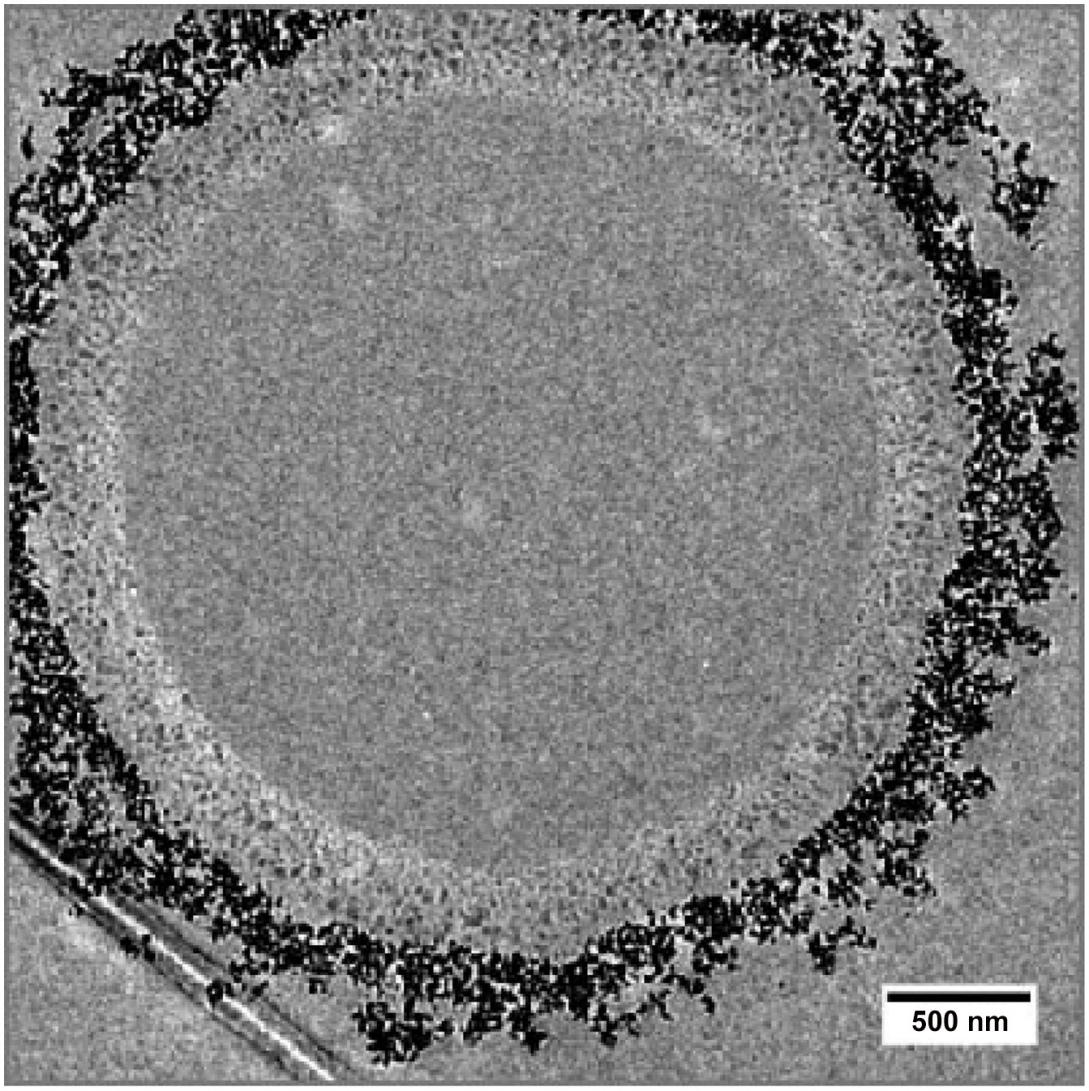
Transmission electron microscopy image of a PVA microbubble. The bubble is coated with iron-oxide nanoparticles, manufactured by Surflay nanotec GmbH, Berlin, Germany. Image courtesy Johan Härmark, School of Technology and Health, KTH Royal Institute of Technology, Sweden.

It is interesting to note that the contribution to the scatter signal that can be attributed to the SPION coating of the PVA bubbles, which contains higher-density material, can be assumed to be negligible. Calculations indicate that the amount of magnetite per bubble distributed over the shell is so low that the overall density difference between the shell and the surrounding water is insignificant compared to the density difference between the air filling and the shell. A detailed analysis of the contributions of different shell layers of varying materials is beyond the scope of this purely experimental work and thus will be addressed in a subsequent publication using a wave-optical simulation framework [34] to evaluate different bubble designs based on the underlying physical principles of the dark-field signal [10, 35]. Additionally, the question whether sub-structuring of the shell into more complex nanoclusters—as it is the case for the PVA bubbles used in this work (see Fig 6)—can be a reason for the dark-field signal increase will be approached in follow-up work.

In this work, we do not provide a quantitative comparison of absolute values of measured dark-field signals, because measuring a reliable quantitative value of the microbubbles in a vial is rendered extremely difficult by the fact that the bubbles float on the surface after already a few seconds if no agitation or fixation is applied. However, since fixation changes the osmotic environment, (partial) destruction of the bubbles and change in the true signal could not be excluded either, thus we refrained from it.

Nevertheless, we can still draw qualitative conclusions from the measurements: The perfusion-simulation experiments, where the strength of the dark-field signal generated by the coated PVA bubbles was measured in a tube against the noise sources of an *ex vivo* animal, support the feasibility of contrast-enhanced dark-field angiography in small animals with adequately designed microbubbles. The PVA microbubbles tested in this work with this given setup are very likely at the edge of detectability when injected into a living animal, depending on the dose tolerance and image acquisition speed. However, they were originally optimized for other imaging modalities such as US, MR and molecular imaging [27, 28], and not specifically designed for dark-field imaging, for example in terms of concentration and size. Also, the sensitivity of the setup used in this work is rather low compared to other grating based x-ray imaging setups, due to the compactness of the interferometer on the gantry. Using a setup with improved sensitivity, which means the ability to resolve smaller refraction angles in the phase signal, for example by smaller grating periods or higher Talbot orders, also a stronger dark-field signal detectability is expected. The application of alternative measurement schemes, for example Fourier-based single-shot image acquisition [36, 37], which also provide phase and dark-field information, but with a much shorter acquisition time, may allow for first-pass bolus imaging before the contrast-agent is diluted beyond detectability. It is safe to say that the imaging protocol can be improved in terms of motion artefacts of skeletal structures and the tubes. However, when translated to the *in vivo* situation, breathing and cardiac motion are an additional challenge. Subtraction imaging and adaptive filtering of the signal proved to be helpful tools for improved signal representation, especially for lower doses of contrast agent, and can surely be optimized for example by widely available non-rigid registration algorithms to tackle motion artifacts [38, 39]. The presented scans have not been optimized in terms of dose, but the accumulated dose of 5.7 mGy is compatible with repeated *in vivo* studies.

## Conclusion

In this work, we showed that SPION-coated PVA microbubbles in a small plastic tube generate a sufficiently large dark-field signal in projection imaging in front of a small-animal background to be detected, even when strongly diluted. Therefore, we anticipate potential of PVA bubbles to be used in dark-field subtraction angiography in small animals.

The complete biochemical design of a dedicated dark-field contrast-agent is beyond the scope of this work and beyond the possibilities of our research laboratory. A future analysis will focus on systematic simulations dedicated to find optimal bubble design parameters such as coating, size and concentration appropriate for various setups laid out for different design energies, as a guideline for preclinical and clinical contrast-agent development.
